# Beverage Consumption among Adults in the Balearic Islands: Association with Total Water and Energy Intake

**DOI:** 10.3390/nu10091149

**Published:** 2018-08-23

**Authors:** Asli Emine Özen, Maria del Mar Bibiloni, Cristina Bouzas, Antoni Pons, Josep A. Tur

**Affiliations:** 1 Department of Gastronomy and Culinary Arts, Reha Midilli Foça Faculty of Tourism, Dokuz Eylül University, Foça-Izmir 35680, Turkey; asli.ozen@deu.edu.tr; 2 Research Group on Community Nutrition and Oxidative Stress, University of the Balearic Islands & CIBEROBN, 07122 Palma de Mallorca, Spain; mar.bibiloni@uib.es (M.d.M.B.); cristinabouvel@gmail.com (C.B.); antonipons@uib.es (A.P.)

**Keywords:** beverage consumption, water consumption, total water intake, total energy intake, adults, Balearic Islands

## Abstract

The paper seeks to describe beverage consumption and examine the association between beverage consumption and total water intake and total energy intake of the adult population in the Balearic Islands. Beverage consumption, total water intake, and total energy intake were obtained by using two 24-h diet recalls from a cross-sectional nutritional survey carried out in the Balearic Islands (*n* = 1386). The contribution of beverages to total water intake and total energy intake were also assessed. Beverages accounted for 65–71% of total water intake and 29–35% of it provided by drinking water. Food moisture contributed 31–37% of total water intake. The mean daily total water intake from all sources was around 2.2 L for men and 1.9 L for women and slightly lower than the proposed adequate intake (AI) recommendations of the European Food Safety Authority (EFSA). The mean total energy intake was 2222 kcal/day and beverages contributed 10.3% of total energy intake for men and 9.5% for women. Energy intake from beverages varied with age. In both sexes, milk was the main beverage contributed to total energy intake. The energy contribution of caloric soft drinks was 1.8% for men and 1.2% for women and energy intake from these beverages was significantly higher among younger adults. Water was the main beverage in the diet, followed by milk and hot beverages. Beverages were mainly consumed in the main meal times (breakfast, lunch, and dinner) in both sexes. The main findings of this study indicate that consumption of sugar-sweetened beverages (caloric soft drinks and commercial fruit juice) is higher among young adults, consumption of alcoholic beverages is higher among males aged 26 and older, and TWI (total water intake) is lower than the EFSA recommendations. These findings may be used to develop effective, healthy eating and drinking policies and campaigns.

## 1. Introduction

Water intake is very essential for human life because water accounts for 50–60% of adult body mass and we need water for the enzymatic and chemical reactions and excretion of metabolic waste from our body [1]. While in our early ancestors’ diet consisted of only drinking water and breast milk [2,3], our beverage choices are vast.

The European Food Safety Authority (EFSA) estimates that 70–80% of total water intake (TWI) comes from drinking water and beverages, while the remaining 20–30% is obtained from food moisture [4]. However, estimates of the Spanish population fall slightly outside these estimations [5]. The contribution of foods and beverages to the TWI for the Spanish population are 32% and 68% [5], respectively. Still, drinking water is the main source of water in the diet of all age groups, and consumption of other beverages varies according to age [6,7,8,9,10,11,12].

Many beverages contribute to total energy intake (TEI). The average energy contribution of beverages to TEI among European countries varies from 7 to 16% (e.g., 7% in Italy [13], 8% in France [14], 12% in Spain [5], and 16% in the UK [15]). Alcoholic beverages are the main contributors to energy intake, followed by milk [14,15].

Recent studies of TWI and beverage consumption and the association between beverage consumption and energy intake among Spaniards have been published [5,12]. A recent study suggested that the population of the Balearic Islands is undergoing a nutrition transition [16]. We, therefore, investigated beverage consumption and TWI, with special attention to the types of beverages consumed and their calorie contribution to total energy intake in a nationally representative sample from the Balearic Islands. 

## 2. Methods

### 2.1. Study Population

Subjects of this study were participants in the OBEX (Obesity and oxidative stress) project which is a population based cross-sectional nutritional survey. The data collection took place between 2009 and 2010. The sample population was derived from residents aged 16–65 years registered in the official population census of the Balearic Islands. The sampling technique included stratification according to municipality size, age, and sex of inhabitants, and the samples were randomization into subgroups, with the Balearic Islands municipalities being the primary sampling units, and individuals within these municipalities comprising the final sample units. The theoretical sample size was set at 1500 individuals and the one specific relative precision of 5% (type I error = 0.05; type II error = 0.10), and the final sample was 1386 (92.4% participation). Pregnant women were excluded from this study. This study was conducted according to the guidelines laid down in the Declaration of Helsinki, and all procedures involving human subjects were approved by the Balearic Islands’ Ethics Committee (Palma de Mallorca, Spain) No. IB/1128/09/PI. Written informed consent was obtained from all subjects and, when they were under 18 years, also from their parents or legal tutors.

### 2.2. General Questionnaire and Anthropometrics

A questionnaire collected the following information: age, marital status, educational level (grouped according to years and type of education: low, <6 years at school; medium, 6–12 years of education; high, >12 years of education), and socioeconomic level (classified as low, medium, and high according to the methodology described by the Spanish Society of Epidemiology) [17].

Information about smoking habits and alcohol consumption was collected and grouped as non-smoker, ex-smoker, smoker, and non-drinker, occasional drinker, daily drinker (1–2 drinks/day), and heavy drinker (more than three drinks/day).

Anthropometric [18] and blood pressure (BP) [19] measurements have been described in full elsewhere. BMI was computed as weight/height^2^ (kg/m^2^) and study participants were categorized as healthy weight (BMI < 24.9 kg/m^2^), overweight (25 kg/m^2^ < BMI < 29.9 kg/m^2^), and obese (BMI ≥ 30 kg/m^2^) [20]. Hypertension was defined as either having a systolic blood pressure (SBP) of ≥140 mmHg or diastolic blood pressure (DBP) of ≥90 mmHg, currently under antihypertensive treatment, or previously diagnosed for hypertension.

### 2.3. Physical Activity Assessment 

Physical activity (PA) was evaluated according to guidelines for data processing and analysis of the International Physical Activity Questionnaire [21] in the short form. The PA levels were estimated by using metabolic equivalents of task (MET). MET scores for different level activities were established based on the Compendium of Physical Activities [22]. On the basis of their total weekly MET scores, the subjects were divided into three groups: “low”, “moderate”, and “high” levels of PA.

### 2.4. Assessment of Beverage Consumption and Energy Intake 

Beverage, food, and energy intakes were assessed by averaging two non-consecutive 24 h dietary recalls. To prevent seasonal variations, 24 h dietary recalls were administered in the warm season (May–September) and in the cold season (November–March). Furthermore, to account for day-to-day intake variability, the two 24-h recalls were administered from Monday to Sunday. Participants reported all foods and beverages consumed throughout the day: breakfast, second breakfast, lunch, afternoon snack, dinner, and outside of meal times. A manual of sets of photographs [23] was used for the estimation of portion size. Well-trained dieticians administered the recalls and verified and quantified the information obtained from the 24 h recalls.

Beverages were categorized into 11 groups; water (tap water and bottled water), full-fat milk, low/non-fat milk (semi-skimmed and skimmed milk), 100% fruit juice (all kinds of natural fruit juice), commercial fruit juice (all kinds of fruit juice sweetened with sugar), caloric soft drinks (all kinds of carbonated soft drinks, sugar added iced tea and energy beverages), diet soda (low calorie carbonated soft drinks), coffee, tea, alcoholic beverages (wine, beer, vodka, whisky, liquor), and other beverages (beer without alcohol, diet milkshake, soy milk, rice milk, oat milk, fermented milk drink with sugar, fermented milk drink, kefir, horchata, chocolate milkshake, isotonic drinks). Total milk included full-fat milk and low/non-fat milk, hot beverages included coffee and tea, and total fruit juice included all kinds of fruit juice were also calculated. TWI and TEI were calculated using a computer program (ALIMENTA^®^, NUCOX, Palma, Spain) based on Spanish [24,25] and European Food Composition Tables [26], and complemented with food composition data available for the Balearic food items [27]. Total water intake was calculated as drinking water plus water from all other beverages and moisture from all foods. Identification of underreporting participants was based on the Goldberg cut-off [28]. Adults whose reported energy intake (EI)/basal metabolic rate (BMR) was <0.9585 were classified as under-reporters (*n* = 328), and they were excluded from the current study.

### 2.5. Statistics

Statistical analyses were performed using SPSS for Windows, version 24.0 (SPSS Inc., Chicago, IL, USA). For descriptive purposes, absolute numbers and percentages of participants were calculated for demographic and lifestyle characteristics and differences tested by *χ*^2^. Average daily beverage consumption, TWI (g/day) and TEI (kcal/day) were calculated and differences across means were evaluated by using analysis of variance. Differences in mean daily water, beverage and energy intake across age groups within sex were assessed by using student *t*-tests with Bonferroni correction for multiple testing. Partial correlations between the consumption of different types of beverages and TWI, water intake from beverages and foods, TEI, energy intake from beverages and foods were adjusted for gender, age, and BMI. For all statistical tests, *p* < 0.05 was taken as the significant level.

## 3. Results

### 3.1. Description of the Survey Sample 

Survey respondents ranged from 16 to 65 years (mean 32 years) (Table 1). Overweight prevalence was higher among men (41%) than women (32%). Men were likely to be single heavy drinker and have hypertension; women reported engaging in less physical activity.

### 3.2. Contribution of Beverages and Food Moisture to Daily Diet 

Daily mean beverage consumption was stratified by gender and age as presented in Table 2. Consumption of full-fat milk, fruit juice, and caloric soft drinks tended to decrease with age in both sexes. Men consumed two times more caloric soft drinks (*p* < 0.001) and alcoholic beverage (*p* < 0.001) than women, while tea (*p* < 0.001) consumption was much higher in women.

The contribution of foods and beverages to daily TWI (g/day) and TEI (kcal/day), by gender and age group, are presented in Table 3. In total, beverages accounted for 71.1% and 65.4% of TWI for men and women, respectively, while the contribution of all foods to TWI was 28.9% for men and 34.6% for women. The mean daily TWI from all sources was around 2.2 L for men and 1.9 L for women. Energy intake from beverages was higher in men than in women and slightly increased with age. The mean TEI of the study population was 2222 (±19) kcal/day, and beverages contributed 9.5% of TEI for females and 10.3% for males. 

### 3.3. Contribution of Beverage Type to Diet

The contribution of beverages to daily water and energy intake by gender and age is presented in Table 4. Water was the most consumed beverage for both sexes and drinking water alone accounted for 31.7% of the TWI for men and 29.2% of the TWI for women. Among other beverages, hot beverages (mainly coffee) and milk were the main sources of TWI for both sexes.

In both males and females, milk was the principal beverage contributor of TEI. Among younger adults, the contribution of commercial fruit juice and caloric soft drinks to TEI was higher, while the energy contribution of alcoholic beverages was higher in middle-aged men. 

### 3.4. Distribution of Beverages during Day

**Figure 1:**
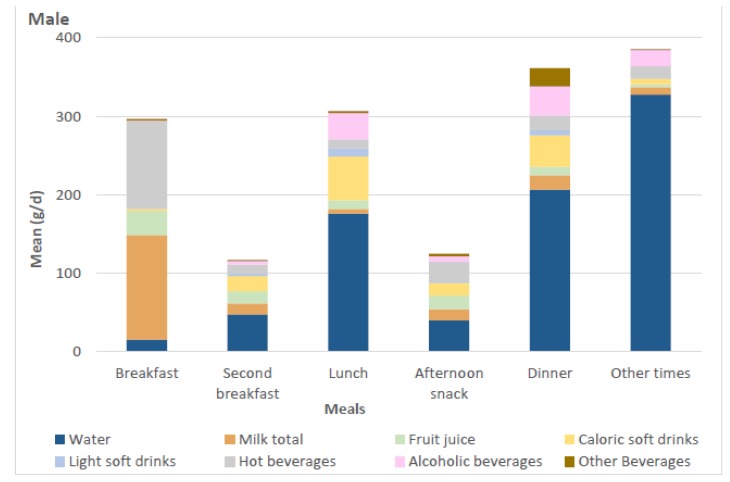

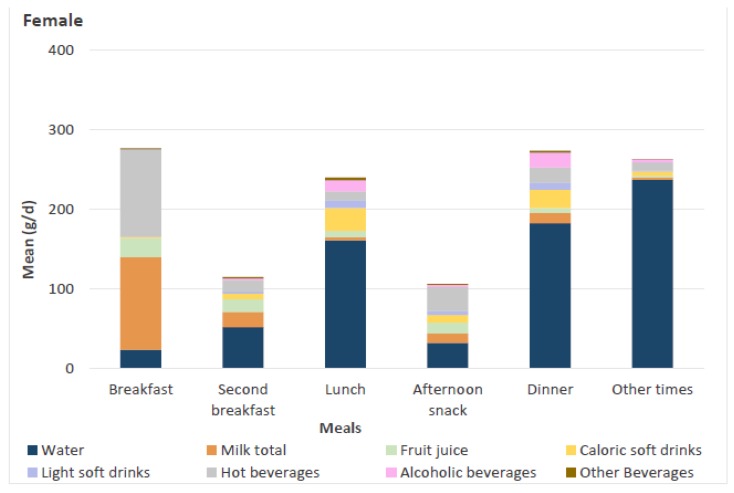
Daily mean beverage consumption during different meals by gender.

Figure 1 shows the mean daily consumption of beverages during each meal time for males and females. Milk, fruit juices, and hot beverages (coffee and tea) were mainly consumed for breakfast in both sexes. Alcoholic beverages were mainly consumed during lunch and dinner. Other beverages were more evenly spread throughout the day, with slightly higher consumption during lunch and dinner. The main part of the water consumption was concentrated in the afternoon, and the highest water intake was observed outside of the meal times.

**Figure 2:**
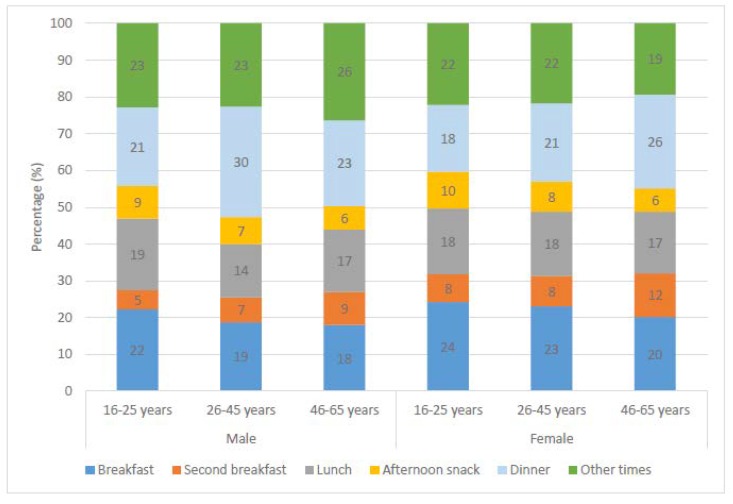
Percentage of total beverage consumption during different meals by gender and age group.

Percentage of total beverage consumption during each meal time is presented in Figure 2. Beverage consumption during dinner was significantly higher in middle-aged men than others, while a higher proportion of older women preferred to consume their beverages during dinner.

In Table 5, we assessed the correlation between the consumption of different types of beverages and TWI, water from beverages and foods, TEI, energy intake from beverages and foods. Water consumption was highly correlated with the total beverage consumption, water intake from beverages and TWI. Consumption of caloric soft drinks was associated with energy intake from foods and beverages and also TEI. In other cases, correlation coefficients were generally unremarkable.

## 4. Discussion

The present study investigated the beverage consumption and TWI and TEI from beverages among Balearic adults. The results show that mean TWI was 2.2 L for men and 1.9 L for women in the study population and slightly lower than the proposed AIs of water, which are 2.5 L for males and 2 L for females by the EFSA [4]. Water recommendations of EFSA are applied only to conditions of moderate environmental temperature and moderate physical activity levels [4]. TWI below the recommended values might be related with the low physical activity level of the study population. We observed that more than half of the study population had a low physical activity level. 

According to the estimation of EFSA, beverages contribute 70–80% of TWI and foods contribute 20–30% of TWI [4]. While men met these estimations, among women water intake from foods was higher, 35%. This difference can be explained with the high vegetable consumption of women as these contain a high amount of food moisture. We observe that women consumed more vegetables (172 g/day) than men did (142 g/day) (data not shown), and this finding is in line with a previous study [29].

In this study, water was the principal beverage, and water accounted for 31% of TWI. In parallel to our findings, drinking water was the main beverage among the entire Spanish population [12,30]. Within other beverages, hot beverages were the main contributor to TWI, followed by milk and caloric soft drinks. 

Energy intake from beverages varied within sex and age-specific groups and mean energy intake of the whole population is 9.8% which was lower than those of the entire Spanish population [12]. Overall, milk is the main beverage, accounting for energy intake, followed by alcoholic beverages and caloric soft drinks. In general, energy contribution of caloric soft drinks was 1.8% for men and 1.2% for women in our study population, but energy intake from these beverages was significantly higher among younger adults, especially in men (2.7% of TEI). In addition to caloric soft drinks, energy intake from commercial fruit juice was higher among younger adults. In comparison with the US population (5.7%) [31], energy intake from caloric soft drinks is lower in the Balearic population, but in view of the adverse health effect of caloric soft drinks [32,33,34], high consumption of these beverages among younger people should be discouraged by health authorities.

Another issue to be raised is the higher energy intake from alcoholic beverages of adults aged 26 and older, particularly men. Many older adults have chronic health conditions, and therefore, they take numerous medications; alcohol intake may interact with these medications [35]. Mean daily alcoholic beverage intake of the study population was below the recommended limits of alcohol and low to moderate alcohol consumption has some health benefits [36,37]. However the body composition changes with age and the amount of total body water decreases, which results in higher blood alcohol concentration in older than younger adults for the same amount of alcohol intake [38]. Close attention needs to be paid by health authorities for identifying high alcohol consumption of older adults since they are at a greater risk of alcohol-related harm than younger drinkers.

Adults in the Balearic Islands consumed more beverages during main meal times. Type of beverages varied between different meal times. Hot beverages, milk, and fruit juices were mainly consumed at breakfast, while water consumption was the lowest during breakfast. Fruit juice and milk have more effects on hunger and satiety than water, and these beverages satisfy thirst like water [39]. This might explain the beverage preferences of the study population and low water consumption during breakfast. Similar to our findings, milk and fruit juices were commonly consumed beverages for breakfast in Norway [40].

Earlier studies have suggested that consumption of sugar-sweetened beverages was related to high levels of energy intake [41,42]. In line with this, consumption of caloric soft drinks was positively correlated with TEI and also energy intake from food. Consumption of caloric soft drinks not only adds empty calories to the diet, but also regular consumption of these energy-dense beverages may affect the food choices and total caloric intake [43].

Some of the strengths of the present study consist of the use of a large and representative sample of Balearic adults. Misreporting of energy intake is an acknowledged problem in all dietary assessment methods [44,45]; Goldberg cut-off methods [28] were applied to exclude under-reporters. Several limitations of the present study need to be mentioned. First, the food and beverage intake and physical activity (IPAQ questionnaire) data are gathered using self-reported questionnaires and might be influenced by recording errors. Estimation of portion size is a usual weakness of self-reported dietary assessment methods; however, we used a manual of sets of photographs to avoid this weakness. Another limitation of the study is its cross-sectional design, which limits conclusions regarding causality. We used two 24 h dietary recalls. Dietary intakes estimated by means of two 24 h dietary recalls are not suitable for determining the usual intake distributions [46]; therefore, we do not attempt to describe the usual intake distributions of daily water intake. We present population means and standard errors for the beverage consumption, TWI, and TEI.

## 5. Conclusions

Although the energy contribution of beverages is low among the Balearic Islands population, there are some issues requiring the attention of health authorities for promoting healthy drinking. The main findings of this study indicate that consumption of sugar-sweetened beverages (caloric soft drinks and commercial fruit juice) is higher among young adults, consumption of alcoholic beverages is higher among males aged 26 and older, and TWI is lower than the EFSA recommendations. These findings may be used to develop effective, healthy eating and drinking policies and campaigns. 

## Figures and Tables

**Table 1:** Socio-demographic and lifestyle characteristics of study population.

Characteristics	Male (*n* = 410)		Female (*n* = 654)		
	*n*	%	*n*	%	*χ* ^2^
**Age (years)**					<0.001
16–25	201	49	228	36	
26–45	150	37	290	45	
46–65	57	14	122	19	
**BMI**					<0.001
Healthy weight	232	57	445	68	
Overweight	143	35	159	24	
Obese	35	9	49	8	
**Marital Status**					0.003
Not married	304	75	426	67	
Married	103	25	213	33	
**Education Level**					ns
Low	136	34	185	29	
Medium	144	36	224	35	
High	124	31	228	36	
**Employment Status**					ns
Low	174	43	240	37	
Medium	40	10	81	13	
High	195	48	324	50	
**Smoking status**					ns
Non smoker	241	61	374	59	
Ex-smoker	48	12	82	13	
Smoker	108	27	174	28	
**Alcohol consumption**					<0.001
Non drinkers	91	23	207	32	
Very occasional drinkers	151	37	263	41	
Daily drinkers	125	31	140	22	
Heavy drinkers	37	9	29	5	
**Hypertension**					0.038
No hypertension	288	71	483	76	
Have hypertension	116	29	149	24	
**Physical Activity**					<0.001
Low	164	41	431	67	
Moderate	132	33	174	30	
High	102	26	43	7	

BMI: Body Mass Index; Not married includes: single, divorced, widowed, and separated. ns: Not statistically significant.

**Table 2:** Mean daily beverage consumption (g/day) by gender and age group.

Beverages	Male					Female				
	16–25	26–45	46–65	Total	*p* Value	16–25	26–45	46–65	Total	*p* Value
	Mean (SE) ^1^	Mean (SE) ^1^	Mean (SE) ^1^	Mean (SE) ^1^		Mean (SE) ^1^	Mean (SE) ^1^	Mean (SE) ^1^	Mean (SE) ^1^	
Water	839.5 (57.3)	817.7 (69.2)	845.3 (110.7)	831.4 (40.7)	ns	709.8 (47.5)	636.0 (38.7)	748.8 (79.3)	688.5 (28.5)	ns
Milk total	228.8 (15.3) ^a^	194.8 (14.6) ^ab^	150.6 (20.1) ^b^	205.6 (9.7) *	0.021	176.4 (9.4)	165.4 (8.2)	181.1 (11.6)	173.4 (5.4)	ns
Full-fat milk	111.8 (11.8) ^a^	85.9 (9.9) ^ab^	59.4 (12.5) ^b^	95.2 (7.1) ***	0.032	94.0 (8.8) ^a^	61.0 (6.1) ^b^	47.7 (9.4) ^b^	70.0 (4.5)	<0.001
Low/non-fat milk	117.0 (13.1)	108.9 (14.0)	91.2 (17.0)	110.4 (8.6)	ns	82.5 (7.9) ^a^	104.4 (7.5) ^ab^	133.4 (10.8) ^b^	103.4 (5.0)	0.001
Fruit juice total	111.4 (14.6) ^a^	83.0 (12.9) ^ab^	49.1 (13.5) ^b^	92.3 (8.8) ***	0.049	96.5 (9.3) ^a^	63.8 (7.0) ^b^	36.0 (8.7) ^b^	70.4 (4.9)	<0.001
100% fruit juice	22.7 (7.3)	28.9 (6.2)	21.1 (8.2)	24.8 (4.4)	ns	20.3 (4.0)	23.7 (3.9)	26.9 (2.7)	23.1 (2.7)	ns
Commercial fruit juice	88.7 (13.2) ^a^	54.1 (11.8) ^ab^	28.1 (9.3) ^b^	67.5 (8.0) *	0.019	76.3 (8.9) ^a^	40.1 (6.1) ^b^	11.1 (4.1) ^c^	47.5 (4.4)	<0.001
Caloric soft drinks	219.5 (24.7) ^a^	84.9 (9.3) ^b^	48.1 (17.4) ^b^	145.4 (14.0) ***	<0.001	129.4 (17.8) ^a^	68.6 (9.7) ^b^	19.6 (6.9) ^c^	79.7 (7.8)	<0.001
Light soft drinks	11.8 (5.5) ^a^	38.9 (9.3) ^b^	10.5 (7.4) ^a^	21.5 (4.5) *	0.014	19.3 (10.6)	36.6 (7.5)	14.4 (6.4)	25.7 (5.1)	ns
Hot beverages total	207.7 (21.8)	181.0 (20.4)	174.2 (26.8)	194.7 (13.6)	ns	219.0 (17.8)	190.1 (11.2)	202.5 (17.7)	202.1 (8.7)	ns
Coffee	194.7 (20.8)	152.3 (19.3)	142.6 (25.8)	173.0 (13.0) ***	ns	193.5 (17.5) ^a^	128.2 (8.7) ^b^	121.6 (13.1) ^b^	150.4 (7.8)	<0.001
Tea	12.9 (5.7)	28.7 (7.1)	31.6 (10.7)	21.7 (4.1) ***	ns	25.4 (5.6) ^a^	61.96 (8.3) ^b^	80.9 (14.4) ^b^	51.7 (5.0)	<0.001
Alcoholic beverages	46.3 (15.8) ^a^	161.0 (37.0) ^b^	179.2 (36.8) ^b^	107.0 (16.7) **	0.001	25.0 (11.0)	51.1 (7.2)	52.4 (10.3)	41.9 (5.4)	ns
Other beverages	3.6 (2.2) ^a^	45.0 (9.5) ^b^	33.4 (13.7) ^b^	22.9 (4.2) ***	<0.001	13.9 (4.5) ^a^	28.72 (4.6) ^ab^	34.2 (8.4) ^b^	24.3 (3.0)	0.031
Total beverage	1668.6 (57.8)	1606.3 (86.5)	1490.4 (115.2)	1620.7 (45.4) *	ns	1390.2 (52.8)	1242.7 (42.0)	1290.9 (79.3)	1307.3 (30.4)	ns

* *p* < 0.05, ** *p* < 0.01, *** *p* < 0.001 (Significantly different from females). Superscript lowercase letters denote significant differences across age group within sex (analysis of variance with Bonferroni correction). ns: not statistically significant. ^1^ Standard Error Means.

**Table 3:** Contribution of food and beverages to total water (g/day) and energy (kcal/day) intake by gender and age group.

	Age Group	Water from Beverages		Water from Foods		Total Water Intake (TWI)	Energy from Beverages		Energy from Foods		Total Energy Intake (TEI)
		Mean (SE) ^1^	%	Mean (SE) ^1^	%	Mean (SE) ^1^	Mean (SE) ^1^	%	Mean (SE) ^1^	%	Mean (SE) ^1^
Male	16–25 years	1604.7 (57.1)	78.4	544.8 (39.3) ^a^	21.6	2149.6 (62.5)	262.7 (13.1)	10.1	2359.1 (48.4) ^a^	89.9	2621.8 (51.0) ^a^
	26–45 years	1542.7 (84.2)	66.8	749.2 (37.9) ^b^	33.2	2291.9 (84.9)	258.3 (15.0)	10.6	2192.6 (51.9) ^b^	89.6	2450.9 (54.6) ^a^
	46–65 years	1438.6 (114.8)	63.1	824.8 (50.8) ^b^	36.9	2263.4 (125.8)	233.8 (18.6)	10.5	1976.8 (59.6) ^c^	89.5	2210.6 (64.1) ^b^
	Total	1558.7 (44.6) ***	71.1	658.4 (44.6)	28.9	2217.1 (47.0) ***	257.1 (8.9) ***	10.3	2244.5 (32.6) ***	89.7	2499.5 (33.9) ***
Female	16–25 years	1343.6 (52.2)	73.5	530.0 (28.7) ^a^	26.5	1873.6 (54.9)	205.7 (9.7)	9.6	1931.6 (33.1) ^a^	90.4	2137.3 (35.0) ^a^
	26–45 years	1202.6 (41.8)	62.1	724.9 (22.8) ^b^	37.9	1925.7 (44.6)	190.2 (7.4)	9.4	1863.1 (28.5) ^a^	90.6	2050.5 (29.3) ^a^
	46–65 years	1249.3 (78.9)	58.8	825.7 (33.1) ^c^	41.2	2074.9 (87.2)	172.6 (10.4)	9.3	1704.4 (34.4) ^b^	90.4	1877.0 (35.4) ^b^
	Total	1264.8 (30.2)	65.4	673.4 (16.2)	34.6	1937.4 (32.4)	192.4 (5.2)	9.5	1857.2 (18.9)	90.5	2048.1 (19.4)
Total		1380.4 (25.7)	68.1	667.5 (14.0)	31.9	2047.4 (27.3)	217.6 (4.8)	9.8	2008.1 (18.0)	90.2	2222.0 (18.9)

*** *p* < 0.001 (Significantly different from females). Superscript lowercase letters denote significant differences across age group within sex (analysis of variance with Bonferroni correction). ^1^ Standard Error Means.

**Table 4:** Contribution of beverages to total water and energy intake by gender and age group.

	Contribution to Water Intake								Contribution to Energy Intake							
	Male				Female				Male				Female			
	Age Group				Age Group				Age Group				Age Group			
	16–25	26–45	46–65	Total	16–25	26–45	46–65	Total	16–25	26–45	46–65	Total	16–25	26–45	46–65	Total
Total intake, Mean(SE) ^1^	2149.6 (62.5)	2291.9 (84.9)	2263.4 (125.8)	2217.1 (47.0) ***	1873.6 (54.9)	1925.7 (44.6)	2074.9 (87.2)	1937.4 (32.4)	2621.8 (51.0) ^a^	2450.9 (54.6) ^a^	2210.6 (64.1) ^b^	2499.5 (33.9) ***	2137.3 (35.0) ^a^	2050.5 (29.3) ^a^	1877.0 (35.4) ^b^	2048.1 (19.4)
From Beverages (%)	78.4	66.8	63.1	71.1	73.5	62.1	58.8	65.4	10.1	10.6	10.5	10.3	9.6	9.4	9.3	9.5
Water (%)	33.3	29.6	32.1	31.7	31.5	27.3	28.5	29.2	0.0	0.0	0.0	0.0	0.0	0.0	0.0	0.0
Milk total (%)	11.1	8.3	6.4	9.4	10	8.5	9.5	9.3	4.7	4.3	3.8	4.5	4.6	4.1	4.4	4.4
Full-fat milk (%)	5.5	3.6	2.6	2.5	5.5	3.2	2.5	2.2	2.7	2.4	1.8	2.5	2.8	1.9	1.8	2.2
Low/non-fat milk (%)	5.6	4.8	3.8	2	4.5	5.3	7.0	2.2	2.0	1.9	2.0	2.0	1.9	2.2	2.6	2.2
Fruit juice total (%)	5.2	3.5	2.2	4.1	5.3	3.1	1.5	3.6	2.0	1.5	1.3	1.7	2.1	1.4	1.0	1.6
100% fruit juice (%)	0.9	1.4	0.8	1.1	1.2	1.2	1.1	1.1	0.4	0.6	0.4	0.5	0.5	0.6	0.7	0.6
Commercial fruit juice (%)	4.3	2.0	2.0	1.3	4.1	1.9	0.4	2.4	1.6	1.0	0.7	1.3	1.8	0.9	0.3	1.1
Caloric soft drinks (%)	10.9	3.6	1.9	6.9	7.1	3.7	0.8	4.3	2.7	1.0	0.8	1.8	1.7	1.0	0.8	1.2
Light soft drinks (%)	0.8	1.8	0.6	1.1	1.0	2.0	1.0	1.4	0.0	0.0	0.0	0.0	0.0	0.0	0.0	0.0
Hot beverages total (%)	11.7	8.8	7.8	10.2	14.2	11.2	10.9	12.2	0.1	0.2	0.3	0.2	0.2	0.4	0.6	0.4
Coffee (%)	11.2	7.4	6.6	9.2	12.8	7.9	6.7	9.4	0.1	0.1	0.2	0.1	0.1	0.2	0.2	0.1
Tea (%)	0.5	1.4	1.3	0.9	1.4	3.3	4.2	2.7	0.1	0.1	0.1	0.1	0.1	0.3	0.4	0.3
Alcoholic beverages (%)	1.8	6.9	8.4	4.6	0.9	2.6	3.1	2.1	0.4	2.6	3.7	1.7	0.5	1.5	1.6	1.2
Other beverages (%)	0.2	1.4	1.1	0.8	0.8	1.5	1.4	1.2	0.2	0.9	0.7	0.5	0.5	1.0	0.9	0.8

*** *p* < 0.001 (Significantly different from females). Superscript lowercase letters denote significant differences across age group within sex (analysis of variance with Bonferroni correction). ^1^ Standard Error Means.

**Table 5:** Partial correlation between the consumption of different types of beverages and TWI, water from beverages and foods, TEI, energy intake from beverages and foods (adjusted for gender, age, BMI, and PA).

Beverages	Total Beverage	Water from Beverages	Water from Foods	TWI	Energy from Beverages	Energy from Foods	TEI
Water	0.837 *	0.853 *	0.107 *	0.851 *	−0.163 *	0.051 *	−0.016
Full-fat milk	0.142 *	0.130 *	−0.166 *	0.047 *	0.374 *	0.092 *	0.186 *
Low/non-fat milk	0.140 *	0.135 *	−0.087 *	0.083 *	0.079 *	0.021	0.033
100% fruit juice	−0.052	−0.057	0.003	−0.055	0.050	−0.070 *	−0.052
Commercial fruit juice	−0.074 *	−0.086 *	−0.014	−0.088 *	0.165 *	0.020	0.070 *
Caloric soft drinks	0.107 *	0.082 *	−0.189 *	−0.019	0.340 *	0.178 *	0.275 *
Light soft drinks	0.070 *	0.076 *	−0.068 *	0.037	−0.077 *	0.009	−0.016
Coffee	0.296 *	0.291 *	−0.567 *	−0.012	0.181 *	0.080 *	0.134 *
Tea	0.059	0.062 *	0.079 *	0.098 *	−0.033	0.031	0.021
Alcoholic beverages	0.191 *	0.179 *	−0.101 *	0.117 *	0.141 *	0.108 *	0.153 *
Other beverages	0.047	0.025	−0.111 *	0.079 *	0.068 *	−0.042	−0.019

* *p* < 0.05 (statistically significant); TWI: Total Water Intake; TEI: Total Energy Intake; BMI: Body Mass Index; PA: Physical Activity.

## Abbreviations

AI	adequate intake
BMR	basal metabolic rate
DBP	diastolic blood pressure
EFSA	European Food Safety Authority
EI	energy intake
FFQ	food frequency questionnaire
IPAQ	International Physical Activity Questionnaire
MD	Mediterranean diet
MET	metabolic equivalent
PA	physical activity
BP	systolic blood pressure
SPSS	Statistical Package for the Social Sciences
TEI	total energy intake;
TWI	total water intake
